# Term Abdominal Pregnancy Revealed by* Amnioperitoneum* in Rural Area

**DOI:** 10.1155/2017/4096783

**Published:** 2017-02-26

**Authors:** Henri Donald Mutarambirwa, Bruno Kenfack, Jovanny Tsuala Fouogue

**Affiliations:** ^1^Saint Vincent de Paul Catholic Hospital, Dschang, Cameroon; ^2^Gynecology and Obstetrics Unit, Dschang District Hospital and University of Dschang, Dschang, Cameroon; ^3^Mbouda District Hospital, Mbouda, Cameroon; ^4^Geneva University Hospitals, Geneva, Switzerland

## Abstract

Abdominal pregnancy (AP) accounts for 1% of ectopic implantations. In sub-Saharan Africa, the high prevalence of sexually transmitted infections explains the increasing frequency of this pathology. In Cameroon it rose from 1/10000 deliveries (1995) to 3.3/10000 (2015). Authors herein report a case of a viable abdominal pregnancy discovered at term during emergency laparotomy for suspected uterine rupture. The 24-year-old G2P0 patient was HIV-positive, under antiretrovirals, though AP exceptionally occurs in HIV patients. She did only two antenatal consultations: her main complaint was abdominal pain but five echographies concluded to normal intrauterine pregnancy. Findings at laparotomy were as follows:* amnioperitoneum,* a live female baby weighing 3.4 kilogrammes without deformities and a placenta deeply inserted on the uterine fundus. Removal of the placenta triggered massive bleeding (2400 milliliters) with shock managed with a tourniquet on the lower uterine segment and fluid resuscitation. Outcome was favourable for the mother and child. Prevention of vertical transmission of HIV was successful with antiretroviral therapy.

## 1. Introduction

Abdominal pregnancy (AP) accounts for 1% of all ectopic pregnancies [1]. It is defined as the total or partial implantation and development of the fertilized egg in the abdominal cavity. This implantation may be primary or secondary to an intraperitoneal abortion of a tubal pregnancy [2].

In sub-Saharan Africa, the high prevalence of sexually transmitted infections (STIs) with subsequent tubal damage may explain the increasing frequency of this pathology [3, 4]. It varies from 1/2256 pregnancies (Congo) to 1/2941 (Nigeria) [3, 5, 6]. In developed countries, AP is rare (1/10000–15000 births); the lowest rate has been reported in Tunisia (1 case/21439) [2]. In Cameroon it rose from 1/10000 deliveries (1995) to 3.3/10000 (2015). Cases diagnosed beyond the fifth month of pregnancy are not rare in low resource settings where AP remains a challenge to the poorly equipped health facilities, leading to high perinatal mortality and maternal morbidity (heavy bleeding, bowel obstruction, and infection) [5, 7–9]. If AP is rare, a term AP with viable foetus in HIV-positive mother is an even rarer phenomenon with very few cases reported in the literature. Though rare, AP is usually associated with high maternal and perinatal morbi-mortality. AP poses serious clinical and imaging challenges. Despite the significant advances in the domain of diagnostic imaging, the likelihood of preoperative diagnosis of AP is still low.

The diagnosis during the second half of pregnancy is suspected in front of painful foetal movements. However, ultrasonographic imaging is the mainstay of diagnosis in resource-poor settings, where laparoscopy and/or magnetic resonance imaging (MRI) are inaccessible. MRI specifies the placental relationship with the abdominal organs while laparoscopy is useful during the first trimester [7].

We report a case of viable term AP in a 24-year-old HIV-positive woman discovered incidentally during a laparotomy indicated for a suspected uterine rupture.

## 2. Case Presentation

The patient was a 24-year-old, gravida 2, para 0, with a past surgical history of right salpingectomy indicated for ruptured ectopic pregnancy 2 years prior to admission. She was admitted with a pregnancy of 39 weeks and 4 days for diffuse abdominal pain of sudden onset with no history of abdominal trauma. She was HIV-positive under tritherapy (Tenofovir, Lamivudine, and Nevirapine) for the past 4 years with good clinical and biological improvement.

She had her menarche at 12, her first sexual intercourse at 16, and 03 sexual partners in her life, and, at 18 years of age, she used combined oral contraceptive drugs. She did two antenatal consultations (ANC) from the 5th month of pregnancy. Antianaemic prevention was done. The pregnancy was marked by recurrent abdominal pain and painful active foetal movements without bowel obstruction or vaginal bleeding. She had 05 documented ultrasounds done by two specialists: gynecologist and radiologist. The first was done at 17th week of gestation. All reported “a singleton viable intrauterine pregnancy with fundal placenta insertion.” Her main complaint on admission was severe diffuse abdominal pain aggravated by the change of position. The patient had reported no prior loss of liquor. On examination, she was conscious with a good general condition. Her blood pressure was 121/83 mmHg, pulse rate at 101 beats per minute, and temperature 37, 6°C. Her conjunctivae were pink, and the cardiovascular and pulmonary exams were unremarkable. The abdomen was distended, deflected to the right, and tender on palpation. There was no uterine contraction. Foetal parts were easily palpable beneath abdominal wall and foetal heart rate (FHR) was 138 beats per minute. On vaginal exam there was no bleeding and the cervix was posteriorly long and closed. The posterior pouch of Douglas was bulging and tender.

The general practitioner on call took the patient to the theatre for emergency laparotomy with a presumed diagnosis of uterine rupture with live foetus.

**Figure 1:**
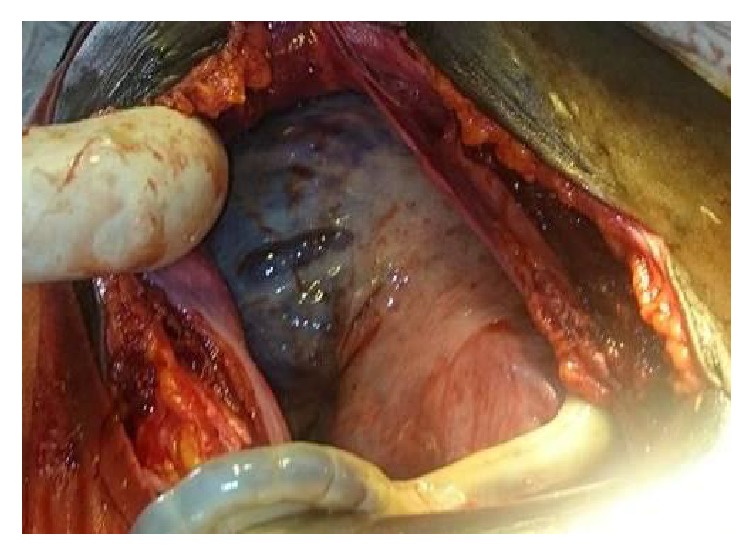
View of the placenta and uterus after extraction of the foetus.

**Figure 2:**
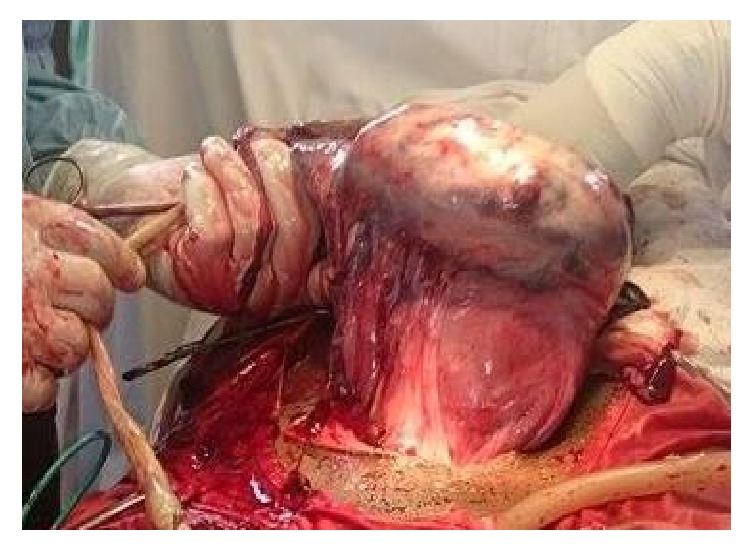
Normal uterus with the placenta implanted on the posterofundal surface.

**Figure 3:**
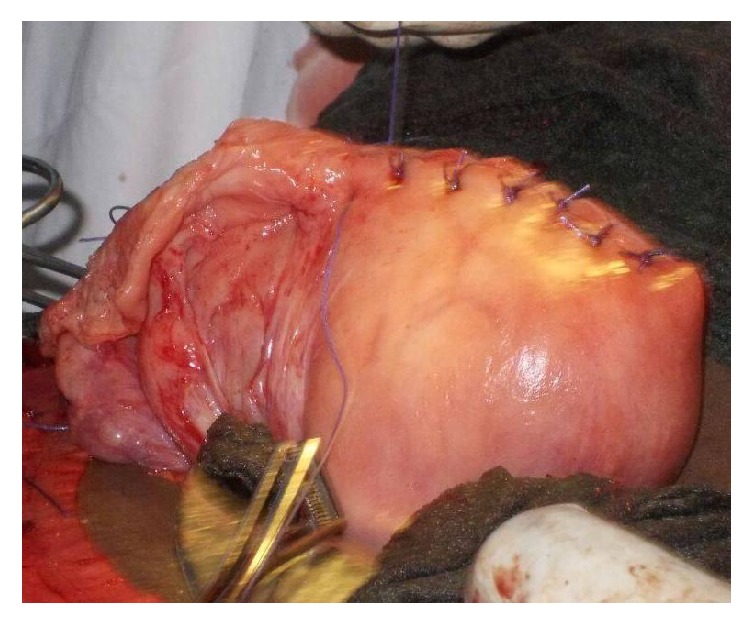
Uterus after placenta removal and uterine suture.

Findings were as follows: 600 milliliters of clear amniotic fluid in the peritoneal cavity; an enlarged uterus to the size of 16 weeks with no sign of rupture and a live (Apgar scores of 7 and 9 at the 1st and 5th minute, resp.) female foetus in the subhepatic area weighing 3400 grammes without deformities. No maternal digestive organs were attached to the foetal adnexae (Figure 1), and the placenta was solely inserted to the uterine fundus (Figure 2). Initial detachment of the placenta led to massive bleeding and hypovolemic shock (blood pressure falling to 50/30 mmHg). Resuscitation was achieved through fluid replacement with 4000 milliliters' saline, oxygenation, and epinephrine. Blood transfusion was not achieved due to lack of compatible blood. A tourniquet (sterile drips set cord) concomitantly placed on the lower uterine segment significantly reduced bleeding and allowed complete removal of the placenta. Uterine repair was done with three layers of interrupted sutures. The left fallopian tube was macroscopically normal (Figure 3). Estimated blood loss was 2400 milliliters and urine output was normal.

Postoperative follow-up was uneventful and the patient was discharged on the ninth postoperative day. She was put on iron therapy for anaemia and was followed up weekly with her baby for six weeks and then monthly for six months. The mother continued with antiretrovirals and the baby was on exclusive breastfeeding and Nevirapine the first six months. The result of the polymerase chain reaction (PCR) test for the detection of HIV at six weeks of age was negative. The mother was counselled on the need for an 18-month intergenesic space and elective caesarean section during next pregnancies. She accepted lactational amenorrhoea method for contraception and pursued with microprogesterone-only pills.

## 3. Discussion

Abdominal pregnancy is extremely rare. Its prevalence is 10 to 25 times higher in blacks than in Caucasians [9]. Its frequency is increasing in Africa because of predisposing factors including low socioeconomic status, making this disease one of the highlights of poverty [2, 10].

The case reported was incidentally discovered at term, despite Antenatal Care (ANC) with serial obstetric echographies. Nassali et al. reported a case of asymptomatic late term AP despite routine ANC demonstrating diagnosis challenge [11, 12]. In a Nigerian series of twenty cases, 55% of AP were not diagnosed before laparotomy [6]. In our case the results of five antenatal echographies made the diagnosis of AP very unlikely on admission.

In our case, surgery was indicated for uterine rupture with a live foetus. Several authors reported similar incorrect preoperative diagnosis: abscess of the Douglas pouch; paralytic ileus; hemoperitoneum; and bowel obstruction [6, 13]. Sometimes misdiagnosed AP is discovered after a failure of induction of term pregnancies [6, 9, 11]. In our case, intraperitoneal spillage of amniotic fluid (due to spontaneous intra-abdominal rupture of membranes) caused peritoneal irritation that led to the wrong diagnosis of uterine rupture. D'cunha suggested that absence of a well constituted amniotic sac and the presence of free amniotic fluid predispose to peritonitis [14]. The abundance of amniotic fluid is among one of the criteria for good foetal prognosis in abdominal pregnancies and might explain why our foetus was alive without deformities [8, 12, 15].

AP is very rare in HIV infected women. Increased likelihood of Sexual Transmitted Infections in HIV-positive women may predispose them to tubal damage and hence to ectopic pregnancy including abdominal pregnancy [16].

Risk factors for AP are those for ectopic pregnancy (EP): subfertility, history of EP, genital malformations, hormonal contraception, tubal surgery, multiple sexual partners, and STIs [3, 17]. Our patient presented all these factors except for malformations.

Abdominopelvic pain is the most common symptom [6, 12, 13]. Our patient had abdominal pain throughout the pregnancy. Other digestive disorders include the following: nausea, postprandial vomiting, bowel obstruction, peritonitis, abnormal foetal lie, and easy palpation of foetal parts beneath maternal skin [4, 14, 15].

Abdominal ultrasound is the key paraclinical exam for AP [7, 12]. It visualizes the foetus in the gestational sac out of the uterus, without uterine wall between the foetus and the bladder. It shows the relationship between the placenta and abdominal organs [7]. Unfortunately echography is an operator-dependent exam that could miss the diagnosis of AP like in our case. Magnetic resonance imaging and laparoscopy may also be useful but are not available in our settings [2, 18].

The management of AP is mainly surgical [2]. A conservative approach may be proposed if diagnosis is made after 20 weeks, provided there is a normal foetal assessment, a placenta implantation site distant from the liver and spleen, a clinical stability of the mother, and a hospital based follow-up with proper information about the risks [18]. In our case the diagnosis was made after emergency laparotomy. The therapeutic challenges were as follows: the young age of the patient and nulliparity and past history of right salpingectomy and the HIV infection. The cataclysmic haemorrhage (2, 4 liters) was to be mastered without compromising her obstetric outcome.

## 4. Conclusion

This case illustrates an AP with two specificities: the diagnosis revealed by the intraperitoneal spillage of amniotic fluid simulating peritonitis from uterine rupture and successful control of bleeding with a tourniquet on the lower uterine segment allowing complete removal of the placenta.
